# The association between statin use and diabetic nephropathy in US adults: data from NHANES 2005 - 2018

**DOI:** 10.3389/fendo.2024.1381746

**Published:** 2024-04-25

**Authors:** Jinjing Guo, Zhibing Jiang, Yiping Xia, Hui Wang, Qun Tang, Bin Meng

**Affiliations:** Medical School, Hunan University of Chinese Medicine, Changsha, Hunan, China

**Keywords:** diabetic nephropathy, statin use, NHANES, diabetes, association

## Abstract

**Background:**

A serious consequence of diabetes is diabetic nephropathy (DN), which is commonly treated by statins. Studies evaluating the effects of statin medication have yielded inconsistent results regarding the potential association with diabetic nephropathy. To manage diabetic nephropathy’s onset and improve the quality of life of patients, it is imperative to gain a comprehensive understanding of its contributing factors.

**Data and methods:**

Our study was conducted using the National Health and Nutrition Examination Survey (NHANES) as well as weighted multivariate logistic regression models to determine the odds ratio (OR) and 95% confidence intervals (95%CI) for diabetic nephropathy. We conducted stratified analyses to examine the impact of statins and the duration of their usage on diabetic nephropathy in different subgroups. A nomogram model and the receiver operating characteristic (ROC) curve were also developed to predict DN risk.

**Results:**

Statin use significantly increased the incidence of DN (OR=1.405, 95%CI (1.199,1.647), p<0.001). Individuals who used statins for 5 to 7 years were more likely to develop diabetic nephropathy (OR=1.472, 95%CI (1.057,2.048), p=0.022) compared to those who used statins for 1-3 years (OR=1.334, 95%CI (1.058,1.682), p=0.015) or <1 year (OR=1.266, 95%CI (1.054,1.522), p = 0.012). Simvastatin has a greater incidence of diabetic nephropathy (OR=1.448, 95%CI(1.177, 1.78), P < 0.001).

**Conclusion:**

Taking statins long-term increases the risk of DN. Statin use is associated with an increased risk of DN. Caution should be exercised when prescribing atorvastatin and simvastatin for long-term statin therapy.

## 1 Introduction

An underlying metabolic disorder characterized by chronic hyperglycemia is diabetes mellitus (1). By 2030, 643 million adults will have diabetes, and by 2045, 783 million will, according to current projections (2). Regrettably, numerous endeavors aimed at managing and mitigating diabetes have proven ineffective owing to the dynamic nature of its consequences. One of the severe complications of diabetes mellitus, diabetic nephropathy (DN) has the potential to develop into end-stage renal disease (3). Additionally, in the early phases of DN, the presence of the condition may increase the risk of cardiovascular disease in diabetic patients, according to one study (4). From 1997 to 2017, DN accounted for one-third of all disability-adjusted life years for individuals with chronic kidney disease worldwide, according to a new report (5). Presently, the primary emphasis of clinical therapies for DN lies in the management of risk factors, including hyperglycemia, hypertension, and proteinuria. These interventions aim to alleviate symptoms and impede the advancement of DN, albeit with restricted effectiveness (6, 7). In this way, a deeper understanding of DN and its associated factors can make a positive difference in its prevention and management, thus improving the prognosis and quality of life of diabetics.

Diabetic patients exhibit a heightened incidence of lipid abnormalities (8). A genus of lipid-lowering medications commonly prescribed for the prevention of atherosclerotic cardiovascular disease (ASCVD) are statins (9, 10). Contradictory results have emerged from clinical trials of statin therapy regarding the potential risk of DN (11–13). Notably, the dosage of statin therapy may play a crucial role in modulating the progression of DN. For instance, an analysis of clinical studies indicated that the utilization of rosuvastatin was linked to a rise in proteinuria and microhaematuria that increased in proportion to the dosage. However, this effect was not observed with other statins (14). In a major insurance claims database analysis, Dormuth et al. discovered that individuals commencing high-potency statins had a 34% higher risk of acute renal damage hospitalization when compared to those on low-potency statins(High-potency statins were defined as atorvastatin ≥20 mg, simvastatin ≥40 mg, or rosuvastatin ≥10 mg; all other statins were defined as low-potency.) (15). Recent research indicates that a 50-week duration of statin use causes lipid deposition and worsens DN (16). Additionally, a recent meta-analysis revealed that the administration of statins did not appear to have a beneficial impact on the occurrence of kidney failure (17, 18).

Nevertheless, several studies appeared to establish a positive correlation between the use of statins and a decreased incidence of DN. For instance, several studies showed that statins have a time-dependent effect on renal function and are more effective in patients with type 2 diabetic nephropathy (19, 20). A study suggests that statins may have a positive effect on COVID-19-associated acute kidney injury (21). Adding atorvastatin to irbesartan provides extra kidney protection in patients with early diabetic nephropathy (22). Potentially attributable to sample variation, these studies’ contradictory findings regarding the correlation between statin use and DN. The benefit of statin use in the treatment of DN remains unknown. Additionally, different statins might contribute to these conflicts.

As a result, our objective is to examine the correlation between statin usage and DN via the seven cycles (2005-2018) of NHANES screening diabetic patients to elucidate the precise impacts of various commonly prescribed statins.

## 2 Methods

### 2.1 Study population

The NHANES is a research initiative that evaluates the health and nutritional condition of individuals residing in the United States. The participants in our study were collected from 7 cycles that occurred between 2005 and 2018. We categorized all participants into two groups: those with diabetes and those without diabetes, based on the widely accepted international diagnostic criteria for diabetes. This study encompassed all individuals diagnosed with diabetes. The criteria for exclusion were as follows: (1) Individuals who are younger than 18 years old or older than or equal to 85 years old, (2) Individuals who are pregnant, (3) Participants who do not have data on plasma creatinine, urinary albumin, urinary creatinine, and statin use.

### 2.2 Data collection

The population’s fundamental data was gathered by proficient individuals, and all experimental measurements were rigorously conducted throughout the procedure by experts in compliance with the technical requirements given on NHANES’ official website. The NHANES website provides the option to download all data and experimental procedures. The experiments were conducted at a laboratory located in Minnesota.

Data was collected on various demographic factors including age, gender, race, education, finance, marital status, smoking habits, alcohol consumption, physical activity levels. Blood pressure and body mass index(BMI), results from biochemical tests such as high-density lipoprotein cholesterol (HDL-C), low-density lipoprotein cholesterol (LDL-C), glycosylated hemoglobin (Hba1c), alanine aminotransferase(ALT), aspartate transaminase(AST), alkaline phosphatase(ALP), albumin(ALB), globulin(GLB), total cholesterol and triglyceride were also included. Individuals who used any form of statin, either on their own or in conjunction with other medications, were classified as statin users.

### 2.3 Definition of diabetic nephropathy

Health measurements are performed in specially-designed and equipped mobile centers, which travel to locations throughout the country. DN is identified at this point in time, according to participants’ urine and blood samples which were collected in the NHANES’s mobile examination centers (MECs). Patients with an estimated glomerular filtration rate (eGFR) below 60 mL × min^−1^ × 1.73 m^−2^, as determined by the Modification of Diet in Renal Disease (MDRD) Study equation, or patients with a urine albumin-creatinine ratio(ACR) equal to or more than 30 mg/g, were diagnosed with DN. The MDRD estimate of kidney function was derived using the formula: 175 × plasma creatinine^−1.154^ × age^−0.203^ (× 0.742 if female; × 1.21 if black) (23).

### 2.4 Definition of statin users

A survey was conducted during the in-home interview to evaluate the utilization of prescribed drugs. Individuals who indicated in the questionnaire that they had previously used statins were categorized as statin users. The duration of statin usage was categorized based on the specific number of days as follows: less than one year (<365 days), one to three years (≥365 days and <1095 days), three to five years (≥1095 days and <1825 days), five to seven years (≥1825 days and <2555 days), seven to ten years (≥2555 days and <3650 days), and more than ten years (≥3650 days).

### 2.5 Diagnosis of diabetes mellitus

Diabetic patients were determined to have diabetes based on self-reports of diabetes, hypoglycemic drugs, or meeting the American Diabetes Association diagnostic criteria (glycosylated hemoglobin ≥ 6.5% or fasting blood glucose > 7.0mmol/L) (24, 25).

### 2.6 Definition of hypertension

We calculated the mean blood pressure from three successive readings taken while the individual was in a relaxed state. The term “hypertension” was defined in the following manner: (1) Mean systolic blood pressure equal to or more than 140 mmHg, (2) Mean diastolic blood pressure equal to or greater than 90 mmHg, (3) Self-reported hypertension, (4) Antihypertensive drug users. The 140/90 mmHg criterion is based on recommendations made by the International Society of Hypertension (26).

### 2.7 Covariable screening

Demographics encompass factors such as age, gender, race, educational level, financial situation, and marital status. Race was classified as Mexican American, Other Hispanic, Non-Hispanic White, Non-Hispanic Black, and Other Race. The education level was categorized into three groups: low (less than a high school diploma), middle (high school graduate or equivalent), and high (college or above). The financial position was categorized into three groups: low, middle, and high, based on the poverty income ratio (PIR ≤ 1, 1<PIR<4, PIR≥4). The marital status was categorized as accompanied, separated, or never married. The body mass index is calculated by dividing an individual’s weight (in kilograms) by the square of their height (in square meters) (27).

The Healthy Lifestyle encompassed data regarding cigarette smoking, alcohol intake, and physical activity. Participants were categorized into two groups based on their self-reported smoking history: Individuals who self-reported having smoked a minimum of 100 cigarettes during their lives, as well as those who smoked fewer than 100 cigarettes. Annual alcohol consumption was classified as “12 cups or more” or “less than 12 cups.” “Average daily physical activity,” “vigorous recreational activity,” and “moderate recreational activity” were utilized to classify physical activity in the questionnaire as either active or inactive. Individuals exhibiting HDL (high-density lipoprotein) levels below 40 mg/dL, total cholesterol levels equal to or beyond 240 mg/dL, triglyceride levels surpassing 200 mg/dL, or LDL levels at or above 160 mg/dL were categorized as having dyslipidemia (28).

The biochemical indicators comprise BUN, Hba1c, HDL, ALB, ALT, AST, ALP, BUN, SCR, Total cholesterol, Triglyceride, LDL, and GLB. Subsequently, all quantities were measured and expressed about internationally recognized standard units.

### 2.8 Statistical methods

Data processing and analysis were conducted using R version 4.3.0 (2023-04-21), in conjunction with the Storm Statistical Platform (www.medsta.cn/software). As shown in Prevention (2023) (29), the NHANES reporting requirements adjust and recognize the NHANES sample weights.

Statistical significance was determined for P values less than 0.05. Continuous variables are depicted through comprehensive sample descriptions accompanied by a 95% confidence interval. Discrete counts and weighted proportions represent categorical variables. The atypical distribution is depicted by the median and the interquartile range (Q1-Q3). The participants were categorized into two groups: the DN group and the non-DN group, based on whether their ACR exceeded 30 or their eGFR was below 60. We employed the “multiple imputation” technique to address the issue of missing covariates, mitigating the potential selection bias that could arise from eliminating participants with incomplete data. We aimed to ascertain whether the presence of DN was influenced by the utilization of statins, in comparison to not using them.

Following the construction of the weighted single-factor and weighted multifactor logistic regression models, four distinct models were generated in accordance with the variable types. Model 1, which is the single-factor logical regression model, does not involve any variable adjustments. Model 2 was modified to account for variables such as gender, race, BMI, and finance. Model 3 incorporated the variables from model 2, along with adjustments for smoking, drinking, physical activity, and dyslipidemia. Model 4 included additional variables compared to model 3, namely Hba1c, HDL, ALB, AST, ALT, ALP, LDL, Triglyceride, Total cholesterol, and GLB.

We conduct a more detailed analysis in the group of patients with diabetes to investigate the impact of different types of statins and the length of treatment on DN. In order to evaluate the strength of the results, weighted stratified logistic regression and subgroup analyses were conducted on each of the subgroups. The duration of statin usage was subsequently converted to a categorical variable. Furthermore, to examine for interaction terms between subgroups during effect correction tests, likelihood ratio tests were applied. The nomogram model was constructed using R. Using receiver operating characteristic curve (ROC), the discriminatory authority of the nomogram model in DN risk detection was assessed.

## 3 Result

### 3.1 Basic information

**Figure 1:**
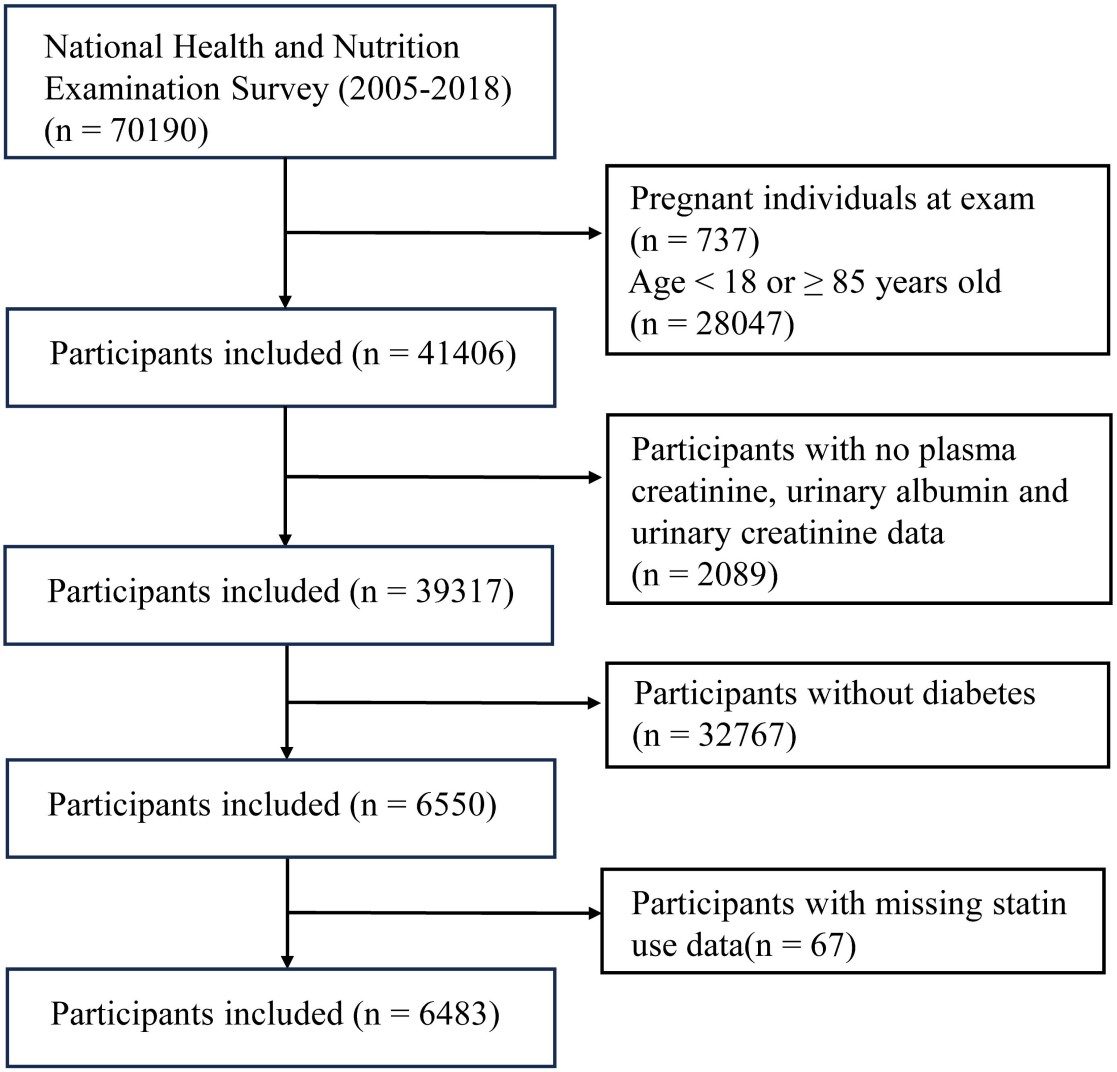
Flowchart of participant selection.

After undergoing screening by the aforementioned stringent criteria (**Figure 1**), a total of 6483 diabetic patients (mean age: 61.14 ± 13.67 years) were selected from the NHANES 2005–2018 population (n = 70,190). The aforementioned groups comprised over 200 million adults residing in the United States. The baseline characteristics are specified in **Supplementary Table 1**. Individuals who presented with DN were found to be older (67.00 (58.00 - 76.00) years), male (53.83%), non-Hispanic white (37.83%), accompanied (87.69%), alcoholics (46.83%), hypertensive (89.83%), possess a high level of education (38.19%), have a moderate financial situation (58.07%), engage in inactive physical activity (74.31%), and utilize statins (53.94%). The glycosylated hemoglobin (7.00 (6.30, 8.20) %, GLB (3.10 (2.80, 3.40) g/dL), ALP (74.00 (60.00, 94.00) g/L), BUN (18.00 (13.00, 25.00) mg/dL), SCR (1.10 (0.83, 1.40) mg/dL), and triglyceride (132.00 (92.00 - 197.00) mg/dL) of patients with DN were all significantly higher than those of patients without DN. The levels of BMI and AST did not differ significantly between the two groups. We also found that in this study, 3,116 participants used statin medication, while 3,367 did not (**Supplementary Table 1**). There were significant differences between the two groups in terms of age, gender, race, marital status, economic status, hypertension, physical activity, diabetic nephropathy, and several biochemical indicators (P<0.05). Specifically, statin users had a higher incidence of diabetic nephropathy (45.03% vs 35.58%, P<0.001).

### 3.2 Univariate regression analysis

The results of univariate logistic regression analysis (**Table 1**) indicated that the following factors were associated with DN: educational situation, finance, smoking, drinking, physical activity, dyslipidemia, GGT, statin use, Hba1c, BUN, SCR, ALP, GLB, and ALB. Individuals with a high level of education or financial resources are at a reduced risk of developing DN compared to those low or middle. Non-smokers have a reduced risk of developing DN in comparison to smokers. Conversely, individuals who consume alcohol, are physically inactive or have dyslipidemia are at an elevated risk of developing DN. A negative correlation was observed between age, Hba1c, BUN, SCR, ALP, and GLB with the incidence of DN. Conversely, there existed a positive correlation between ALB and the incidence of DN. Furthermore, pharmacological indicators (specifically, statin use) demonstrated a significant association with the exacerbation of DN symptoms (OR=1.32, 95%CI (1.147,1.52), p<0.001) in comparison to those who did not use statins. This association carries a 32% increased risk of developing DN.

**Table 1:** Univariate analysis of association between factors of statin use and diabetic nephropathy.

Variable	Diabetic nephropathy	
	OR (95%CI)	P-value
Age	1.042(1.037,1.048)	<0.001
Gender		
Male	1	
Female	0.985(0.856,1.132)	0.829
Race		
Mexican American	1	
Other Hispanic	0.901(0.705,1.151)	0.404
Non-Hispanic White	1.076(0.904,1.281)	0.41
Non-Hispanic Black	1.034(0.868,1.232)	0.706
Other Race - Including Multi-Racial	1.008(0.785,1.296)	0.949
NA	2.138(0.58,7.88)	0.254
BMI	1(0.991,1.008)	0.914
Finance		
Low	1	
Medium	0.895(0.761,1.053)	0.18
High	0.592(0.476,0.736)	<0.001
NA	0.764(0.589,0.991)	0.043
Cigarette smoking		
No	1	
Yes	1.183(1.028,1.361)	0.019
NA	1.764(0.531,5.858)	0.354
Alcohol drinking		
No	1	
Yes	0.732(0.626,0.857)	<0.001
NA	0.732(0.6,0.894)	0.002
Physical activity		
No	1	
Yes	0.732(0.627,0.855)	<0.001
NA	0.64(0.211,1.94)	0.43
Dyslipidemia		
No	1	
Yes	1.303(1.129,1.504)	<0.001
Statin use		
No		
Yes	1.32(1.147,1.52)	<0.001
Hba1c	1.148(1.105,1.194)	<0.001
HDL	0.998(0.992,1.003)	0.44
ALB	0.929(0.911,0.948)	<0.001
AST	1.001(0.998,1.004)	0.504
ALT	0.998(0.992,1.004)	0.452
ALP	1.006(1.002,1.009)	<0.001
Total cholesterol	0.999(0.998,1.001)	0.461
Triglyceride	1(1,1)	0.252
LDL	1(0.999,1.002)	0.77
GLB	1.758(1.528,2.023)	<0.001

NA, Missing; BMI, Body Mass Index; HDL, High Density Lipoprotein; ALB, Albumin; ALT, Alanine Aminotransferase; AST, Aspartate Transaminase; ALP, Alkaline Phosphatase; LDL, Low Density Lipoprotein; GLB, Globulin.

### 3.3 Multivariate regression analysis

Utilizing weighted multivariate logistic regression, the relationship between statin use and DN was investigated. **Table 2** details the relationship between statin use and DN, and four logistic regression models were developed, with the effect value expressed as odds ratio and 95% confidence interval. Depending on the magnitude of the effect size, patients treated with statins may have a relatively increased risk of developing DN. The unadjusted analysis (model 1) revealed a statistically significant association between statin use and DN (OR 1.32; p<0.001). This association corresponds to a 32% escalation in the risk of DN among patients who were prescribed statins. The effect value in model 2, which was marginally adjusted, was (OR=1.349, 95%CI (1.168,1.558), p<0.001). This value indicated that patients taking statins had a 34.9% increased risk of developing DN. The effect value in further adjusted model 3, denoted as (OR=1.351, 95%CI (1.165,1.566), p<0.001), suggested that patients undergoing statin treatment faced a 35.1% increased risk of developing DN. The effect value in fully adjusted model 4 was (OR=1.405, 95%CI (1.199,1.647), p<0.001), indicating that the use of statins significantly increased the risk of DN by 40.5% in comparison to those who did not use statins.

**Table 2:** Multivariate analysis of statin use and related factors of diabetic nephropathy.

Variable	Model 1		Model 2		Model 3		Model 4	
	OR (95%Cl)	P-value	OR (95%Cl)	P-value	OR (95%Cl)	P-value	OR (95%Cl)	P-value
**Statin use**	1.32(1.147,1.52)	p<0.001	1.349(1.168,1.558)	p<0.001	1.351(1.165,1.566)	p<0.001	1.405(1.199,1.647)	p<0.001
P for trend	p<0.001		p<0.001		p<0.001		p<0.001	
Statin types								
ATORVASTATIN	1.347(1.109,1.636)	0.003	1.383(1.136,1.683)	0.001	1.407(1.152,1.719)	0.001	1.443(1.168,1.784)	0.001
FLUVASTATIN	1.225(0.203,7.38)	0.825	1.011(0.154,6.644)	0.991	1.233(0.196,7.757)	0.823	1.407(0.194,10.208)	0.736
LOVASTATIN	1.335(0.902,1.976)	0.148	1.341(0.901,1.998)	0.148	1.358(0.905,2.038)	0.139	1.428(0.955,2.134)	0.082
SIMVASTATIN	1.354(1.122,1.634)	0.002	1.382(1.14,1.674)	0.001	1.365(1.122,1.661)	0.002	1.448(1.177,1.78)	p<0.001
PITAVASTATIN	1		1		1		1	
PRAVASTATIN	1.218(0.882,1.682)	0.23	1.229(0.889,1.699)	0.211	1.221(0.882,1.689)	0.229	1.253(0.896,1.752)	0.187
ROSUVASTATIN	1.295(0.901,1.862)	0.162	1.327(0.925,1.902)	0.124	1.331(0.928,1.908)	0.12	1.385(0.946,2.027)	0.094
None	1		1		1		1	
Duration								
<1 year	1.194(1.004,1.418)	0.045	1.225(1.029,1.459)	0.022	1.218(1.021,1.453)	0.028	1.266(1.054,1.522)	0.012
1 to 3 years	1.261(1.017,1.562)	0.034	1.298(1.045,1.611)	0.018	1.305(1.047,1.626)	0.018	1.334(1.058,1.682)	0.015
3 to 5 years	1		1		1		1	
5 to 7 years	1.484(1.096,2.01)	0.011	1.509(1.108,2.056)	0.009	1.523(1.112,2.086)	0.009	1.472(1.057,2.048)	0.022
7 to 10 years	0.893(0.555,1.437)	0.642	0.901(0.553,1.468)	0.677	0.886(0.537,1.462)	0.637	0.951(0.56,1.615)	0.852
>10 years	1.481(0.877,2.501)	0.142	1.457(0.865,2.454)	0.157	1.519(0.894,2.581)	0.122	1.609(0.951,2.722)	0.077
None	1		1		1		1	

Model 1: Non-adjusted.

Model 2: Gender, Race, BMI, Finance.

Model 3: Model 2+ Smoking, Drinking, Physical activity, Dyslipidemia.

Model 4: Model 3 + Hba1c, HDL, ALB, AST, ALT, ALP, LDL, Triglyceride, Total cholesterol, GLB.

The reference for statin types was participants without statin use (None).

### 3.4 Subgroup analysis and the nomogram model

**Figure 2:**
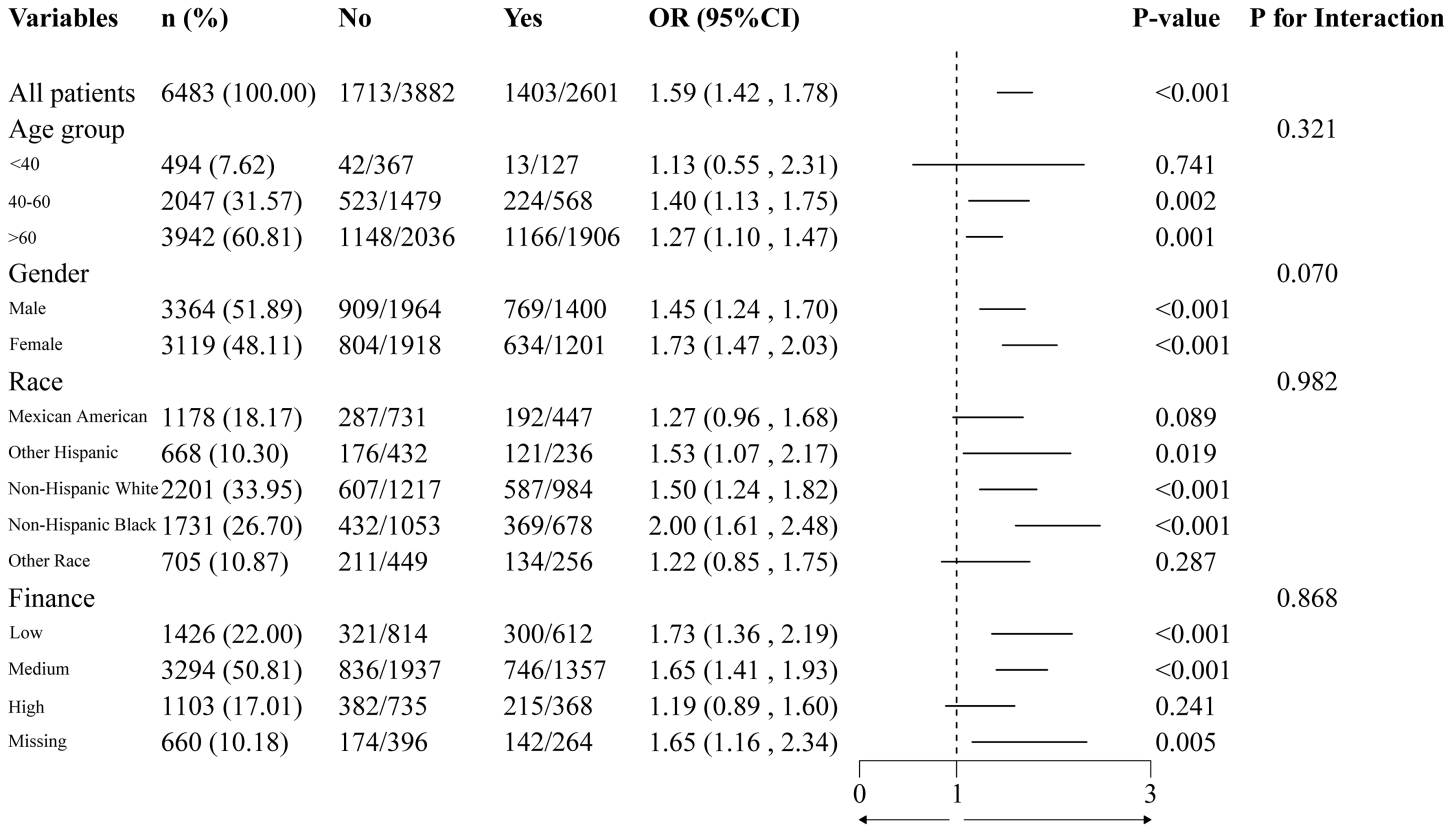
Forest plot of statin use and diabetic nephropathy.

To ensure the results’ stability, subgroup and sensitivity analyses were performed. We examined whether DN and statin use differed with regard to age, gender, race and finance. The findings indicated that the association between statin use and DN remained consistent across all subgroups (**Figure 2**), and no interaction was observed (P > 0.05).

**Figure 3:**
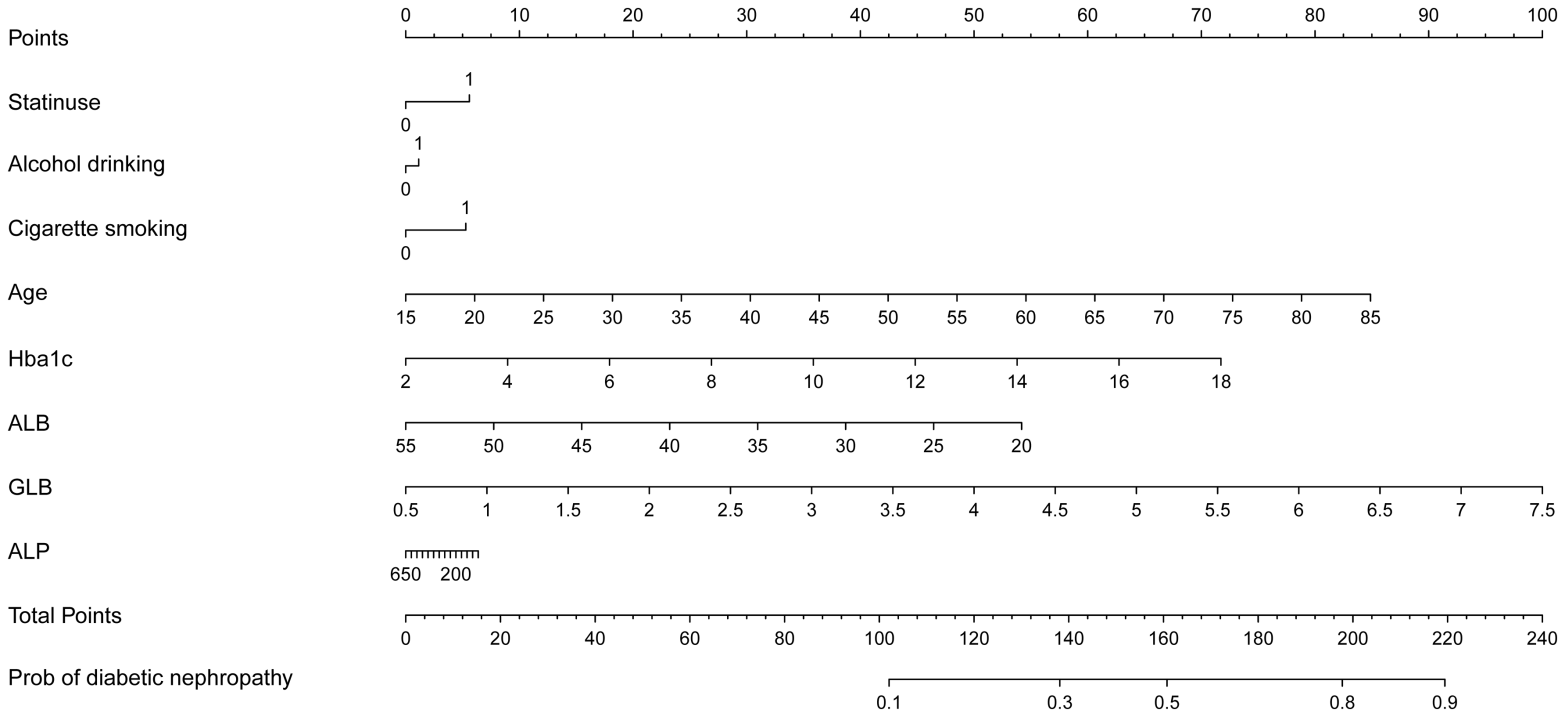
Nomogram model established for predicting the risk of diabetic nephropathy.

**Figure 4:**
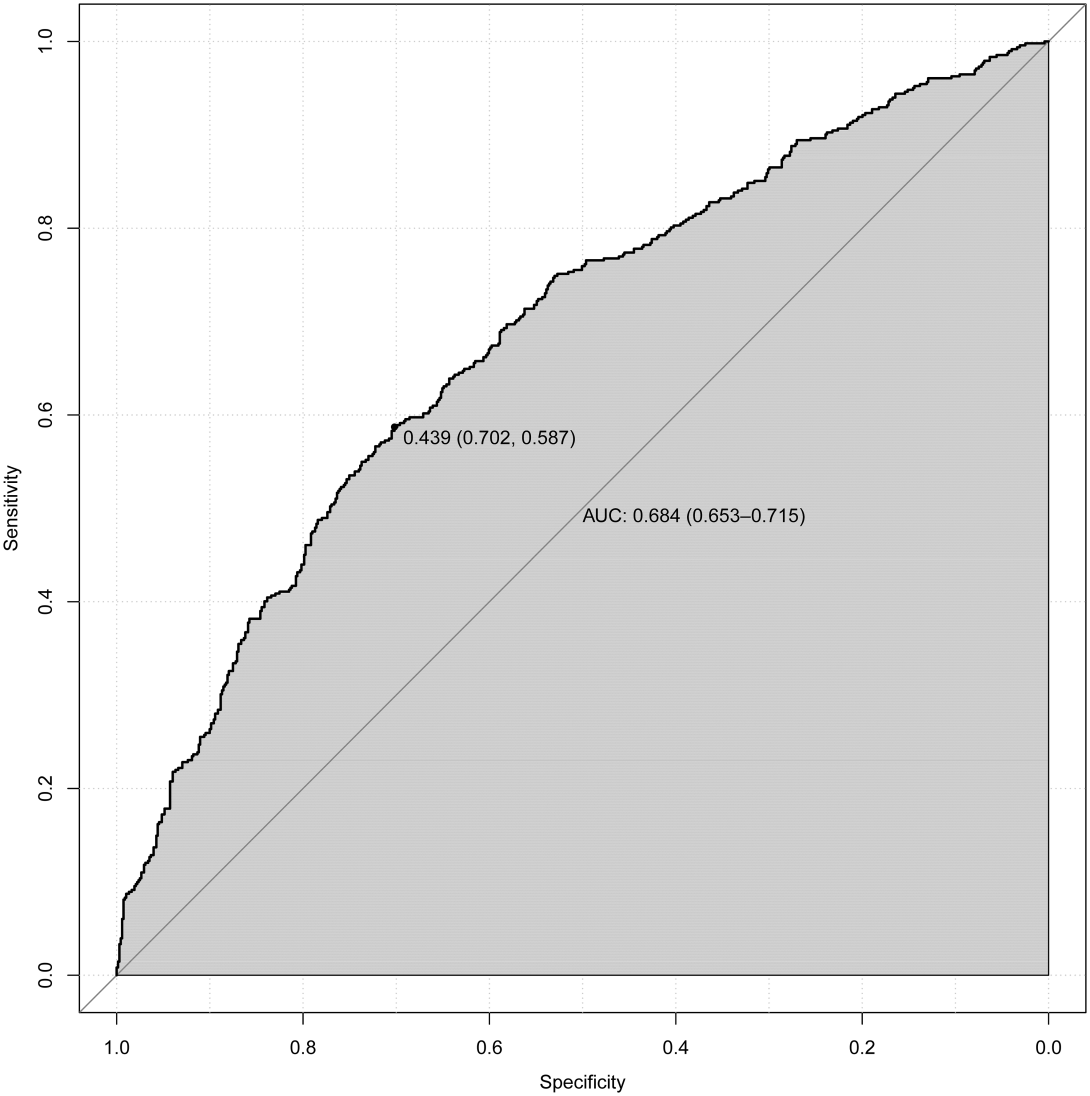
ROC curve for evaluating the diagnostic power of the nomogram model in this study.

Upon adjusting for model 4, individuals who had used statins for 5 to 7 years had a significantly higher and more pronounced risk (OR=1.472, 95%CI (1.057,2.048), p=0.022) of developing DN compared to those who had used them for < 1 year (OR=1.266, 95%CI (1.054,1.522), p = 0.012), 1 to 3 years (OR=1.334, 95%CI(1.058,1.682), p=0.015) or 3 to 5 years of statin use (OR=1). However, as the duration of use increases (7 to 10 years, p = 0.852; or > 10 years, p = 0.077), this association gradually weakens and becomes statistically insignificant after more than 7 years of use. According to NHANES, the five most frequently prescribed statins in the entire sample from 2005 to 2018 (**Supplementary Table 1**) were simvastatin, atorvastatin, pravastatin, rosuvastatin, and lovastatin. A significant risk of developing DN was associated with atorvastatin (OR=1.443, 95%CI (1.168,1.784), p = 0.001). Furthermore, simvastatin, the most frequently prescribed statin, exhibited noteworthy and even the most pronounced ascending effects on the development of DN (OR=1.448, 95%CI (1.177, 1.78), P<0.001) (**Table 2**). We selected variables (age, statin use, alcohol drinking, cigarette smoking, Hba1c, ALB, GLB, ALP) that were more clinically and statistically significant to construct a nomogram model (**Figure 3**). The discriminatory capacity of the nomogram model developed in this study was confirmed by the area under the curve (AUC) of 68.4% (95% CI: 65.3%–71.5%) as shown in the ROC curve (testing) results (**Figure 4**).

## 4 Discussion

The majority of scientists believe that statins protect DN, according to an extensive body of clinical trials and fundamental research. A meta-analysis revealed that statins can enhance renal function indicators in DN therapy, lowering inflammation and preserving the kidney (30). According to a randomized clinical trial, atorvastatin appears to have stronger renoprotective effects in the chronic renal disease group (31). However, it is crucial to highlight that the duration of all trials and fundamental research was brief. Furthermore, there is a lack of data regarding the long-term administration of statins for diabetes from trials that have lasted over a decade (32).

As of yet, the impact of statins on the progression of DN is contentious. The available experimental and clinical data indicate that dyslipidemia has the potential to accelerate the advancement of chronic kidney disease (CKD) by impacting the renal microvasculature and promoting the activation of intracellular signaling pathways associated with inflammation and fibrosis (33). Nevertheless, the impact of lipid-lowering drugs on the advancement of CKD remains uncertain. Numerous studies have documented in recent years that statins can impair glucose metabolism and exacerbate insulin resistance; users of statins have an increased risk of developing type 2 diabetes at the commencement of the disease (34). The potential adverse effects of statin therapy cast doubt on the efficacy of statin use in the treatment of diabetes (35). Recent research has elicited apprehension on the potential renal adverse effects associated with some statins, particularly when administered at elevated dosages (36).

Recent years have seen the confirmation of a number of large-scale randomized controlled trials (RCTs) that statin therapy does not inhibit the progression of kidney disease within five years in a wide range of patients with CKD (37). Moreover, certain case studies have demonstrated that statins like Rosuvastatin has the potential to cause interstitial nephritis (38). More significantly, the use of statins that have a high efficacy in lowering CHOL levels may elevate the likelihood of developing severe renal failure (39). These findings serve as a reminder that the extent to which the beneficial effects of prolonged statin use on DN outweigh its detrimental effects remains uncertain.

We first compared and contrasted the effects of statins in patients with DN. Among the 6483 participants who did not take statins, we discovered that statin use was significantly associated with an increased risk of developing DN. We devised four logical regression models to examine the correlation between the use of statins and DN to account for potential confounding variables The effect value in model 4 is 1.405 (1.199,1.647) (p<0.001). This indicates that statin-treated patients have a 1.32-fold increased risk of developing DN compared to non-statin-treated patients. Concurrently, we examined the correlation between the duration of statin use and DN and categorized statin users according to this criterion. The results indicated that individuals who had been taking statins for a longer period (5 to 7 years) had a significantly increased risk of developing DN (**Table 2**). But this association gradually weakens and becomes statistically insignificant after more than 7 years of use (7 to 10 years, p = 0.852 or > 10 years, p = 0.077). Furthermore, upon validating the findings across age, gender, race, and finance subgroups, we observed that they remained constant without interaction across all subgroups (**Figure 2**). Simvastatin and atorvastatin were associated with a significantly increased risk of DN. The red points show an example in **Supplementary Figure 1** that, for 80-year-old diabetic participant with statin use, alcohol drinking, cigarette smoking, Hba1c (8%), ALB (35g/L), GLB (3g/dL) and ALP (100IU/L), the risk of DN increased by 73%.

Several studies may provide a partial explanation for the mechanisms underlying statin use and DN. Statins, a class of drugs, act as competitive and potent inhibitors of HMGCR, a microsomal enzyme that facilitates the rate-determining conversion of HMG-CoA to mevalonate in LDL cholesterol, thereby reducing cholesterol levels in the blood (40). Various statins exhibit comparable effectiveness in reducing LDL cholesterol levels in relation to their primary purpose of mitigating the likelihood of atherosclerotic cardiovascular events. This result offers compelling evidence that the main impact of statins is the reduction of low-density lipoprotein cholesterol (41). However, it remains uncertain whether there are variations among statins in terms of their effects on the kidneys. A growing body of evidence suggests that statins may cause disruptions in insulin resistance; these findings suggest the possibility of therapeutic adverse effects for diabetes (42). An experiment indicated that inflammatory stress enhanced resistance to statins and boosted intracellular CHOL production by improving HMG-CoA-R activity (43). Chronic inflammation caused by stress triggers the production of cholesterol within cells, which interferes with the normal control of HMG-CoA-R in the kidneys through SCAP. This disruption results in a condition known as “renal statin resistance” (44). An animal experiment demonstrated that the administration of statins over a prolonged period in mice resulted in a significant upregulation of CD36 expression in the kidneys (16). CD36 is a multifunctional receptor that facilitates the internalization of oxidized lipids and long-chain fatty acids. Lipid accumulation, inflammatory signaling, energy reprogramming, apoptosis, and renal fibrosis are among the many functions it performs (45). It could be quite some time before these effects become apparent. Hence, it partially facilitates the development of DN. Therefore, it is hypothesized that diabetic patients may face an elevated risk to their renal function as a result of prolonged statin use. This potential risk highlights the importance of close monitoring and management of renal function in diabetic patients who are prescribed statins.

The limited sample size of participants in this study who used statins for more than 7 years may explain the results showing a loss of statistical significance after more than 7 years of use. Further prospective research is warranted to validate and generalize these findings to a larger and more diverse population. It is essential to fully understand the impact of statin use on renal function in diabetic patients in order to optimize their treatment and improve their overall health outcomes.

## 5 Limitations

Several limitations apply to this investigation. NHANES datasets do not contain information regarding the dosage of statins, even though this is a critical variable that can significantly impact their efficacy or adverse effects. Since a portion of the data was collected via questionnaire and memory, the survey may be subject to recall bias. Statin users and non-users may have potential confounding factors at baseline, such as differences in the risk of cardiovascular disease. We recognize that the adjustments made to the model may still be insufficient to completely eliminate confounding bias. The NHANES database’s inclusion was constrained in our study by its exclusive focus on normal populations, thereby excluding certain special populations and other countries.

## 6 Conclusion

In conclusion, the use of statins was associated with an increased risk of DN in adults in the United States; furthermore, the risk of DN is increased with long-term statin use. Atorvastatin and simvastatin should be used with caution in long-term treatment with statins.

## Data availability statement

The original contributions presented in the study are included in the article/**Supplementary Material**. Further inquiries can be directed to the corresponding author.

## Ethics statement

The studies involving humans were approved by National Center for Health Statistics Ethics Review Board. The studies were conducted in accordance with the local legislation and institutional requirements. The participants provided their written informed consent to participate in this study.

## Author contributions

JG: Conceptualization, Data curation, Software, Writing – original draft, Writing – review & editing. ZJ: Data curation, Writing – review & editing. YX: Software, Writing – review & editing. HW: Software, Writing – review & editing. QT: Funding acquisition, Investigation, Methodology, Project administration, Writing – review & editing. BM: Methodology, Writing – review & editing.

## Supplementary material

Supplementary Figure 1 Nomogram model based on the significant factors screened by logistic regression.
