# Supplementary material for: The association between statin use and diabetic nephropathy in US adults: data from NHANES 2005 - 2018
Source: Front Endocrinol (Lausanne). 2024 Apr 25;15:1381746. doi: 10.3389/fendo.2024.1381746 (PMC11079199; doi:10.3389/fendo.2024.1381746)
Supplement: Supplementary file 1 [file Table_1.docx]

Supplementary Material

# Supplementary Table 1. Demographic characteristics describe whether diabetic nephropathy occurs.

| **Variable** | **Total (n = 6483)** | **Whether diabetic nephropathy occurs** | | **Statistic** | **P-value** |
| --- | --- | --- | --- | --- | --- |
|  |  | **No (n = 3882)** | **Yes (n = 2601)** |  |  |
| Age, M (Q₁, Q₃) | 63.00 (52.00 - 72.00) | 60.00 (50.00 - 68.00) | 67.00 (58.00 - 76.00) | Z=-20.964 | <0.001 |
| BMI, M (Q₁, Q₃) | 31.16 (27.17 - 36.22) | 31.13 (27.26 - 36.24) | 31.20 (27.02 - 36.20) | Z=-0.281 | 0.779 |
| Use Days, M (Q₁, Q₃) | 292.00 (146.00 - 365.00) | 274.00 (146.00 - 365.00) | 292.00 (146.00 - 365.00) | Z=-1.256 | 0.209 |
| Hba1c, M (Q₁, Q₃) | 6.80 (6.20 - 7.90) | 6.70 (6.20 - 7.70) | 7.00 (6.30 - 8.20) | Z=-7.804 | <0.001 |
| HDL, M (Q₁, Q₃) | 46.00 (39.00 - 56.00) | 46.00 (39.00 - 56.00) | 45.00 (38.00 - 55.00) | Z=-3.373 | <0.001 |
| ALB, M (Q₁, Q₃) | 41.00 (39.00 - 43.00) | 42.00 (39.00 - 44.00) | 41.00 (38.00 - 43.00) | Z=-10.309 | <0.001 |
| AST, M (Q₁, Q₃) | 23.00 (19.00 - 28.00) | 23.00 (19.00 - 28.00) | 22.00 (18.00 - 29.00) | Z=-1.401 | 0.161 |
| ALT, M (Q₁, Q₃) | 21.00 (16.00 - 30.00) | 22.00 (17.00 - 31.00) | 20.00 (15.00 - 28.00) | Z=-8.414 | <0.001 |
| GLB, M (Q₁, Q₃) | 3.00 (2.70 - 3.30) | 3.00 (2.70 - 3.30) | 3.10 (2.80 - 3.40) | Z=-8.894 | <0.001 |
| ALP, M (Q₁, Q₃) | 73.00 (59.00 - 90.00) | 72.00 (58.00 - 88.00) | 74.00 (60.00 - 94.00) | Z=-4.352 | <0.001 |
| BUN, M (Q₁, Q₃) | 15.00 (11.00 - 19.00) | 13.00 (11.00 - 16.00) | 18.00 (13.00 - 25.00) | Z=-28.088 | <0.001 |
| SCR, M (Q₁, Q₃) | 0.90 (0.73 - 1.11) | 0.82 (0.70 - 0.96) | 1.10 (0.83 - 1.40) | Z=-32.197 | <0.001 |
| Total Cholesterol, M (Q₁, Q₃) | 180.00 (153.00 - 213.00) | 183.00 (155.00 - 213.00) | 175.00 (149.00 - 211.00) | Z=-4.862 | <0.001 |
| Triglyceride, M (Q₁, Q₃) | 126.00 (89.00 - 185.00) | 125.00 (87.00 - 179.00) | 132.00 (92.00 - 197.00) | Z=-3.596 | <0.001 |
| LDL, M (Q₁, Q₃) | 100.00 (77.00 - 128.00) | 104.00 (79.00 - 130.00) | 96.00 (73.25 - 123.75) | Z=-4.756 | <0.001 |
| Gender, n (%) |  |  |  | χ²=6.521 | 0.011 |
| Male | 3364 (51.89) | 1964 (50.59) | 1400 (53.83) |  |  |
| Female | 3119 (48.11) | 1918 (49.41) | 1201 (46.17) |  |  |
| Race, n (%) |  |  |  | χ²=32.884 | <0.001 |
| Mexican American | 1178 (18.17) | 731 (18.83) | 447 (17.19) |  |  |
| Other Hispanic | 668 (10.3) | 432 (11.13) | 236 (9.07) |  |  |
| Non-Hispanic White | 2201 (33.95) | 1217 (31.35) | 984 (37.83) |  |  |
| Non-Hispanic Black | 1731 (26.7) | 1053 (27.13) | 678 (26.07) |  |  |
| Other Race | 705 (10.87) | 449 (11.57) | 256 (9.84) |  |  |
| Education, n (%) |  |  |  | χ²=24.254 | <0.001 |
| Low | 2251 (34.79) | 1268 (32.72) | 983 (37.88) |  |  |
| Middle | 1527 (23.6) | 906 (23.38) | 621 (23.93) |  |  |
| High | 2692 (41.61) | 1701 (43.90) | 991 (38.19) |  |  |
| Marriage, n (%) |  |  |  | χ²=8.781 | 0.012 |
| Accompanied | 5589 (86.64) | 3316 (85.93) | 2273 (87.69) |  |  |
| Separated | 246 (3.81) | 141 (3.65) | 105 (4.05) |  |  |
| Never married | 616 (9.55) | 402 (10.42) | 214 (8.26) |  |  |
| Finance, n (%) |  |  |  | χ²=27.188 | <0.001 |
| Low | 1426 (24.49) | 814 (23.35) | 612 (26.19) |  |  |
| Medium | 3294 (56.57) | 1937 (55.57) | 1357 (58.07) |  |  |
| High | 1103 (18.94) | 735 (21.08) | 368 (15.75) |  |  |
| Alcohol drinking, n (%) |  |  |  | χ²=5.835 | 0.016 |
| <12 drinks/year | 1851 (37.39) | 1059 (36.02) | 792 (39.40) |  |  |
| ≥12 drinks/year | 3099 (62.61) | 1881 (63.98) | 1218 (60.60) |  |  |
| Cigarette smoking, n (%) |  |  |  | χ²=16.501 | <0.001 |
| Less than 100 | 3265 (50.5) | 2036 (52.57) | 1229 (47.42) |  |  |
| At least 100 | 3200 (49.5) | 1837 (47.43) | 1363 (52.58) |  |  |
| Hypertension, n (%) |  |  |  | χ²=224.813 | <0.001 |
| No | 1229 (19.13) | 966 (25.18) | 263 (10.17) |  |  |
| Yes | 5194 (80.87) | 2871 (74.82) | 2323 (89.83) |  |  |
| Physical activity, n (%) |  |  |  | χ²=14.474 | <0.001 |
| Inactive | 4634 (71.7) | 2708 (69.96) | 1926 (74.31) |  |  |
| Active | 1829 (28.3) | 1163 (30.04) | 666 (25.69) |  |  |
| Dyslipidemia, n (%) |  |  |  | χ²=17.005 | <0.001 |
| No | 3986 (61.48) | 2466 (63.52) | 1520 (58.44) |  |  |
| Yes | 2497 (38.52) | 1416 (36.48) | 1081 (41.56) |  |  |
| Statin use, n (%) |  |  |  | χ²=60.094 | <0.001 |
| No | 3367 (51.94) | 2169 (55.87) | 1198 (46.06) |  |  |
| Yes | 3116 (48.06) | 1713 (44.13) | 1403 (53.94) |  |  |
| Statin drug, n (%) |  |  |  | - | 0.466 |
| ATORVASTATIN | 1110 (35.62) | 599 (34.97) | 511 (36.42) |  |  |
| FLUVASTATIN | 7 (0.22) | 3 (0.18) | 4 (0.29) |  |  |
| LOVASTATIN | 237 (7.61) | 139 (8.11) | 98 (6.99) |  |  |
| SIMVASTATIN | 1163 (37.32) | 627 (36.60) | 536 (38.20) |  |  |
| PITAVASTATIN | 2 (0.06) | 2 (0.12) | 0 (0.00) |  |  |
| PRAVASTATIN | 341 (10.94) | 197 (11.50) | 144 (10.26) |  |  |
| ROSUVASTATIN | 256 (8.22) | 146 (8.52) | 110 (7.84) |  |  |
| t: t-test; SD: standard deviation; χ²: Chi-square test; BMI, Body Mass Index; HDL, High Density Lipoprotein; ALB, Albumin; ALT, Alanine Aminotransferase; AST, Aspartate Transaminase; ALP, Alkaline Phosphatase; LDL, Low Density Lipoprotein; GLB, Globulin; HbA1c, Hemoglobin A1c; BUN, Blood Urea Nitrogen; SCR, Serum Creatinine. | | | | | |
