# Supplementary material for: The association between statin use and diabetic nephropathy in US adults: data from NHANES 2005 - 2018
Source: Front Endocrinol (Lausanne). 2024 Apr 25;15:1381746. doi: 10.3389/fendo.2024.1381746 (PMC11079199; doi:10.3389/fendo.2024.1381746)
Supplement: Supplementary file 2 [file Table_2.docx]

Supplementary Material

# Supplementary Table 2. Demographic characteristics describe statin users and nonusers.

| **Variable** | **Total (n = 6483)** | **Whether statin was used** | | **Statistic** | **P-value** |
| --- | --- | --- | --- | --- | --- |
|  |  | **Yes (n = 3116)** | **No (n = 3367)** |  |  |
| Age, Mean ± SD | 61.14 ± 13.67 | 65.46 ± 10.92 | 57.13 ± 14.70 | t=26.03 | <.001 |
| BMI, Mean ± SD | 32.42 ± 7.62 | 32.24 ± 7.27 | 32.58 ± 7.92 | t=-1.75 | 0.080 |
| Gender, n(%) |  |  |  | χ²=9.25 | 0.002 |
| Male | 3364 (51.89) | 1678 (53.85) | 1686 (50.07) |  |  |
| Female | 3119 (48.11) | 1438 (46.15) | 1681 (49.93) |  |  |
| Race, n(%) |  |  |  | χ²=65.48 | <.001 |
| Mexican American | 1178 (18.17) | 479 (15.37) | 699 (20.76) |  |  |
| Other Hispanic | 668 (10.30) | 297 (9.53) | 371 (11.02) |  |  |
| Non-Hispanic White | 2201 (33.95) | 1194 (38.32) | 1007 (29.91) |  |  |
| Non-Hispanic Black | 1731 (26.70) | 801 (25.71) | 930 (27.62) |  |  |
| Other Race | 705 (10.87) | 345 (11.07) | 360 (10.69) |  |  |
| Education, n(%) |  |  |  | χ²=2.88 | 0.237 |
| Low | 2251 (34.79) | 1050 (33.76) | 1201 (35.74) |  |  |
| Middle | 1527 (23.60) | 741 (23.83) | 786 (23.39) |  |  |
| High | 2692 (41.61) | 1319 (42.41) | 1373 (40.86) |  |  |
| Marriage, n(%) |  |  |  | χ²=66.12 | <.001 |
| Accompanied | 5589 (86.64) | 2793 (89.86) | 2796 (83.64) |  |  |
| Separated | 246 (3.81) | 113 (3.64) | 133 (3.98) |  |  |
| Never married | 616 (9.55) | 202 (6.50) | 414 (12.38) |  |  |
| Finance, n(%) |  |  |  | χ²=27.88 | <.001 |
| Low | 1426 (24.49) | 621 (22.18) | 805 (26.63) |  |  |
| Medium | 3294 (56.57) | 1582 (56.50) | 1712 (56.63) |  |  |
| High | 1103 (18.94) | 597 (21.32) | 506 (16.74) |  |  |
| Alcohol drinking, n(%) |  |  |  | χ²=0.05 | 0.819 |
| ≥12 drinks/year | 3099 (62.61) | 1477 (62.77) | 1622 (62.46) |  |  |
| <12 drinks/year | 1851 (37.39) | 876 (37.23) | 975 (37.54) |  |  |
| Cigarette smoking, n(%) |  |  |  | χ²=17.55 | <0.001 |
| Yes | 3200 (49.50) | 1626 (52.20) | 1574 (46.99) |  |  |
| No | 3265 (50.50) | 1489 (47.80) | 1776 (53.01) |  |  |
| Hypertension, n(%) |  |  |  | χ²=433.80 | <0.001 |
| Yes | 5194 (80.87) | 2839 (91.43) | 2355 (70.98) |  |  |
| No | 1229 (19.13) | 266 (8.57) | 963 (29.02) |  |  |
| Physical activity, n(%) |  |  |  | χ²=29.07 | <0.001 |
| Active | 1829 (28.30) | 782 (25.16) | 1047 (31.21) |  |  |
| Inactive | 4634 (71.70) | 2326 (74.84) | 2308 (68.79) |  |  |
| Dyslipidemia, n(%) |  |  |  | χ²=97.36 | <0.001 |
| Yes | 2497 (38.52) | 1007 (32.32) | 1490 (44.25) |  |  |
| No | 3986 (61.48) | 2109 (67.68) | 1877 (55.75) |  |  |
| Diabetic nephropathy, n(%) |  |  |  | χ²=60.09 | <0.001 |
| Yes | 2601 (40.12) | 1403 (45.03) | 1198 (35.58) |  |  |
| No | 3882 (59.88) | 1713 (54.97) | 2169 (64.42) |  |  |
| HbA1c, Mean ± SD | 7.34 ± 1.75 | 7.26 ± 1.52 | 7.41 ± 1.93 | t=-3.38 | <0.001 |
| HDL, Mean ± SD | 48.18 ± 14.41 | 48.61 ± 13.89 | 47.79 ± 14.85 | t=2.24 | 0.025 |
| ALB, Mean ± SD | 41.03 ± 3.56 | 41.16 ± 3.47 | 40.92 ± 3.64 | t=2.59 | 0.010 |
| AST, Mean ± SD | 26.37 ± 20.14 | 25.06 ± 16.91 | 27.56 ± 22.64 | t=-4.93 | <0.001 |
| ALT, Mean ± SD | 26.76 ± 27.66 | 24.80 ± 31.73 | 28.55 ± 23.17 | t=-5.25 | <0.001 |
| GLB, Mean ± SD | 3.06 ± 0.51 | 3.00 ± 0.48 | 3.12 ± 0.52 | t=-9.43 | <0.001 |
| ALP, Mean ± SD | 77.58 ± 30.99 | 76.15 ± 30.15 | 78.89 ± 31.68 | t=-3.48 | <0.001 |
| BUN, Mean ± SD | 16.48 ± 8.41 | 17.99 ± 8.92 | 15.09 ± 7.66 | t=13.62 | <0.001 |
| SCR, Mean ± SD | 1.02 ± 0.72 | 1.09 ± 0.80 | 0.96 ± 0.63 | t=7.29 | <0.001 |
| ACR, Mean ± SD | 155.78 ± 703.76 | 168.12 ± 702.55 | 144.40 ± 704.78 | t=1.34 | 0.181 |
| eGFR, Mean ± SD | 84.71 ± 30.46 | 77.75 ± 28.15 | 91.08 ± 31.10 | t=-17.66 | <0.001 |
| Total Cholesterol, Mean ± SD | 185.67 ± 47.52 | 168.37 ± 39.99 | 201.49 ± 48.34 | t=-29.49 | <0.001 |
| Triglyceride, Mean ± SD | 161.06 ± 159.37 | 153.93 ± 154.75 | 167.36 ± 163.12 | t=-2.39 | 0.017 |
| LDL, Mean ± SD | 105.02 ± 38.21 | 89.41 ± 31.99 | 119.00 ± 37.92 | t=-23.53 | <0.001 |

t: t-test; SD: standard deviation; χ²: Chi-square test; BMI, Body Mass Index; HDL, High Density Lipoprotein; ALB, Albumin; ALT, Alanine Aminotransferase; AST, Aspartate Transaminase; ALP, Alkaline Phosphatase; LDL, Low Density Lipoprotein; GLB, Globulin; HbA1c, Hemoglobin A1c; BUN, Blood Urea Nitrogen; SCR, Serum Creatinine; ACR, Albumin to Creatinine Ratio; eGFR, Estimated Glomerular Filtration Rate.
