# Supplementary figures and images for: The association between statin use and diabetic nephropathy in US adults: data from NHANES 2005 - 2018
Source: Front Endocrinol (Lausanne). 2024 Apr 25;15:1381746. doi: 10.3389/fendo.2024.1381746 (PMC11079199; doi:10.3389/fendo.2024.1381746)

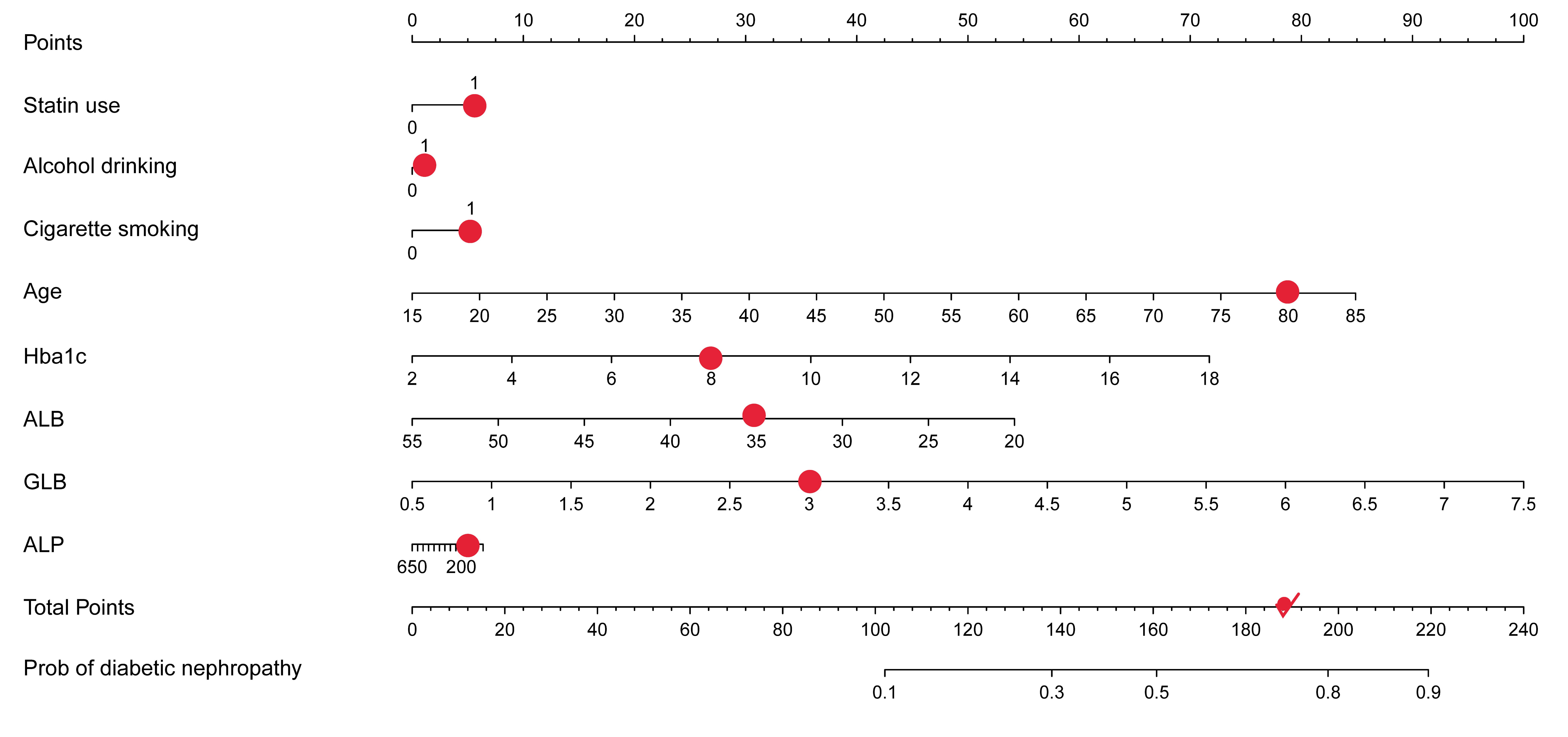

Supplement: Supplementary Figure 1 — Nomogram model based on the significant factors screened by logistic regression. [file Image_1.tif]
